# Associations of serum carotenoid concentrations and fruit or vegetable consumption with serum insulin-like growth factor (IGF)-1 and IGF binding protein-3 concentrations in the Third National Health and Nutrition Examination Survey (NHANES III)

**DOI:** 10.1017/jns.2016.1

**Published:** 2016-03-08

**Authors:** Anja Diener, Sabine Rohrmann

**Affiliations:** Division of Chronic Disease Epidemiology, Epidemiology, Biostatistics and Prevention Institute, University of Zurich, Zurich, Switzerland

**Keywords:** Carotenoids, Insulin-like growth factor-1, Insulin-like growth factor binding protein-3, Third National Health and Nutrition Examination Survey, IGF, insulin-like growth factor, IGFBP, insulin-like growth factor binding protein, NHANES III, Third National Health and Nutrition Examination Survey

## Abstract

Dietary intervention may alter the insulin-like growth factor (IGF) system and thereby cancer risk. In a qualitative review, eleven of twenty studies showed a link between one or more carotenoids, vegetable or fruit intake and the IGF system, however, with partly contrary findings, such that no firm conclusion can be drawn. Therefore, we evaluated associations between serum carotenoid concentrations or the intake of fruits and vegetables with IGF-1, IGF binding protein (BP)-3 and their molar ratio (IGF-1:IGFBP-3) within the Third National Health and Nutrition Examination Survey (NHANES III, 1988–1994). In our analysis, we included 6061 NHANES III participants and used multivariable-adjusted linear regression models. IGF-1 concentrations were significantly positively associated with serum concentrations of lycopene, β-carotene, α-carotene, β-cryptoxanthin and lutein/zeaxanthin in men and women. Statistically significant positive associations were observed for serum concentrations of α-carotene and lutein/zeaxanthin and intake of fruits with serum IGFBP-3 concentrations in women, but not in men. The IGF-1:IGFBP-3 molar ratio was significantly positively associated with serum concentrations of lycopene, β-carotene and α-carotene in men and with β-carotene in women. In conclusion, dietary interventions with carotenoids, fruits and vegetables may affect the IGF system, although the direction of these effects is currently unclear.

The primary role of the human growth hormone insulin-like growth factor (IGF) axis is the regulation of both prenatal and postnatal growth^(^^1^^,^^2^^)^, and IGF binding proteins (IGFBP) are essential modulators of the biological actions of IGF^(^^3^^)^. Besides the regulation of normal growth and ageing, the IGF system is also involved in carcinogenesis^(^^4^^)^. Circulating concentrations of IGF system components are determined by both genetic factors (40–60 %, polymorphisms, imprinting) as well as dietary and lifestyle factors (e.g. diet, smoking, physical activity and others)^(^^5^^)^.

Carotenoids, such as α-carotene, β-carotene and β-cryptoxanthin, are known as provitamin A^(^^6^^)^. Dietary β-carotene is present at relatively high concentrations in carrots and yellow and green leafy vegetables; α-carotene is present in carrots and red palm oil; and β-cryptoxanthin is found in sweet red pepper, oranges, tangerines and papaya^(^^7^^)^. Six species of carotenoids comprise about 60–70 % of the total carotenoid pool in human plasma^(^^8^^,^^9^^)^. The potential of carotenoids to modulate immune responses has been demonstrated *in vivo* and in cell models^(^^10^^)^. Besides their antioxidant activity^(^^11^^)^, investigators have proposed that carotenoids affect cell–cell communications^(^^12^^)^ and have effects on membrane structure and signal transduction pathways^(^^10^^)^. Large numbers of epidemiological studies have explored a possible association between carotenoid intake and reduced risk of CVD and cancer^(^^13^^–^^18^^)^, such as breast cancer^(^^19^^)^, nasopharyngeal carcinoma^(^^20^^)^ or urothelial cell carcinoma^(^^21^^,^^22^^)^.

Lycopene, an acyclic non-provitamin A carotene, has received considerable attention for its possible role in cancer prevention, especially prostate cancer^(^^23^^–^^25^^)^. Lycopene is a tomato-derived substance, but is also present in watermelon, pink grapefruit, guava, papaya and apricots^(^^26^^)^ and accounts for about 50 % of the carotenoids found in human blood; thus, it is the predominant carotenoid. It is a natural fat-soluble pigment and the most potent singlet oxygen quencher and free radical scavenger among all natural carotenoids^(^^27^^)^. Therefore, lycopene is thought to decrease cancer risk through a reduction in oxidative damage^(^^28^^)^. In animal models, effects of lycopene on the endocrine IGF system, i.e. increased serum IGFBP-3 and decreased serum IGF-1:IGFBP-3 ratio, have been described^(^^29^^)^. Lycopene may inhibit activation of the IGF-1 receptor^(^^30^^)^, alter IGF-1-stimulated cell proliferation *in vitro*^(^^30^^,^^31^^)^, induce cell cycle arrest^(^^26^^,^^28^^)^, modulate intercellular communication via gap junction mechanisms^(^^32^^,^^33^^)^, and have strong provitamin A activity via RAR-retinoid-X receptors (RXR) signalling pathways^(^^34^^–^^36^^)^.

Based on the observations that both carotenoids and components of the IGF system have been found to be associated with cancer risk in some studies and potential associations between lycopene, as the most abundant carotenoid, and the IGF system in *in vitro* and *in vivo* studies, it was the aim of our study to examine the associations between circulating levels of carotenoids and fruit and vegetable consumption and serum concentrations of IGF-1, IGFBP-3 and their molar ratio in a cross-sectional US study.

## Methods

### Study population and data collection

The Third National Health and Nutrition Examination Survey (NHANES III) is a nationally representative sample of civilian non-institutionalised individuals conducted by the National Center for Health Statistics of the Centers for Disease Control and Prevention from 1988 to 1994^(^^37^^)^. This cross-sectional study was designed to collect the health and nutrition information of Americans aged 2 months and older through a structured household interview, serum collection and a physical examination in a mobile examination centre. In NHANES III, 39 695 persons were recruited^(^^37^^)^. The protocols for the conduct of NHANES III were approved by the Institutional Review Board of the National Center for Health Statistics, US Centers for Disease Control and Prevention. Informed consent was obtained from all participants.

In NHANES III, a FFQ over a 1-month reference period was to assess habitual diet, and we computed total fruit (four food items) and total vegetable (eleven food items) consumption based on the FFQ information. However, since only consumption frequency, but not portion sizes, has been assessed, we were not able to adjust for energy, macro- or micronutrient intake in our analysis.

### Ascertainment of carotenoids, insulin-like growth factor-1 and insulin-like growth factor binding protein-3

Serum concentrations of α-carotene, β-carotene, β-cryptoxanthin, lycopene and lutein/zeaxanthin were determined using isocratic HPLC-based methods with detection at three different wavelengths (300, 325 and 450 nm; Waters HPLC System). Because these methods do not discriminate lutein from zeaxanthin, the combined concentration of lutein and zeaxanthin is used in analyses. Median interassay CV were 9·4 % for α-carotene, 7·0 % for β-carotene, 8·7 % for β-cryptoxanthin, 7·7 % for lycopene and 11·0 % for lutein/zeaxanthin^(^^38^^)^. IGF-1 and IGFBP-3 concentrations were assessed in serum samples of NHANES III participants, 20+ years old (*n* 6061) from the NHANES III study population, who had participated in a morning examination^(^^39^^)^. Individuals who participated in the morning session provided a blood sample after a mean overnight fast of approximately 11 h. Serum IGF-1 concentrations were available for 2742 men and 3316 women. Three persons were excluded with missing data. Serum samples were sent to the Diagnostic Systems Laboratories (DSL) in Webster, TX, which used the IGF-1 enzyme-linked immunosorbent assay (DSL 10-5600) and the IGFBP-3 immunoradiometric assay (DSL 6600) to measure serum concentrations of these biomarkers. Data for storage history and quality control for the 6061 serum samples from NHANES III have been reported in another study^(^^39^^)^. The molar ratio of IGF-1:IGFBP-3 was computed as an indicator of free IGF-1 concentration^(^^40^^)^.

### Statistical analysis

For the characterisation of the study population, medians and interquartile ranges of all continuous parameters and percentages of all categorical parameters by sex were computed. Linear regression was used to examine the associations of fruit and vegetable consumption and serum concentrations of carotenoids (independent variables) with serum concentration of IGF-1, IGFBP-3 and their molar ratio (IGF-1:IGFBP-3) (dependent variables). Because serum carotenoid concentrations were not normally distributed in the study population, these values were transformed using the natural logarithm. Thus, we computed geometric mean concentrations of carotenoids by quintiles of IGF-1 and IGFBP-3, separately for men and women. We decided to stratify by sex *a priori* because IGF-1 and IGFBP-3 concentrations differ between men and women. For fruit and vegetable consumption, which were normally distributed, we computed arithmetic means by sex. In the linear regression models, we controlled for age (continuous), race/ethnicity (non-Hispanic white, non-Hispanic black, Mexican American, other), smoking status (current, former or never smoker), alcohol consumption (non-consumer, up to once per week, less than daily but more than once/week, at least once per d), BMI (continuous) and serum cholesterol concentration (continuous). These variables were chosen *a priori* based on a literature review. Trend tests were performed by assigning to each individual the value 1 to 5 for the concentration/consumption category (1–5) into which the subject fell. We modelled this term as a continuous variable and the coefficient was evaluated by the Wald test. All tests were two-sided; *P* values <0·05 were considered to be statistically significant.

All statistical analyses were performed using SUDAAN^(^^41^^)^ as implemented in SAS v. 9.1 (SAS Institute) software and weighted to take into account over-sampling, refusal, selection probabilities and differences from the general US population^(^^37^^)^. The protocols for the conduct of NHANES III were approved by the Institutional Review Board of the National Center for Health Statistics, US Centers for Disease Control and Prevention. Informed consent was obtained for all participants^(^^25^^)^.

## Results

Of the 2742 men included in this analysis, 77·6 % were non-Hispanic white, 9·5 % non-Hispanic black and 5·3 % Mexican-American; of the 3316 women, 76·5 % were non-Hispanic white, 11·3 % non-Hispanic black and 4·8 % Mexican-American. Table 1 shows baseline characteristics of study participants and the baseline median concentrations of carotenoids, IGF-1 and IGFBP-3. Men were younger and had higher BMI than women. Men were more likely to be current smokers and current drinkers of alcohol than women. Serum concentrations of all carotenoids but serum lycopene tended to be higher in women than in men. Serum IGF-1 concentrations tended to be higher and IGFBP-3 lower in men than in women.

**Table 1.** Baseline characteristics of study participants in the Third National Health and Nutrition Examination Survey (NHANES III) by sex (Percentages, medians and interquartile ranges or mean values and 95 % confidence intervals)

Variables	Men (%)	Women (%)
Sex	46·6	53·4
Race/ethnicity		
Non-Hispanic white	77·6	76·5
Non-Hispanic black	9·5	11·3
Mexican-American	5·3	4·8
Other	7·6	7·4
Alcohol consumption		
Never	35·4	51·9
Up to once/week	18·0	21·3
Less than daily but more than once/week	31·5	21·1
Daily	15·1	5·7
Smoking status		
Never smoker	36·6	54·6
Former smoker	33·2	21·3
Current smoker	30·2	24·1
Age (years)		
Median	39·8	40·8
Interquartile range	29·4–54·2	30·3–56·7
BMI (kg/m^2^)		
Median	25·9	25·1
Interquartile range	23·5–29·2	21·8–29·7
Serum lycopene (μmol/l)		
Geometric mean	0·40	0·37
95 % CI	0·38, 0·41	0·36, 0·38
Serum β-carotene (μmol/l)		
Geometric mean	0·23	0·32
95 % CI	0·22, 0·24	0·30, 0·33
Serum α-carotene (μmol/l)		
Geometric mean	0·05	0·07
95 % CI	0·05, 0·06	0·06, 0·07
Serum β-cryptoxanthin (μmol/l)		
Geometric mean	0·12	0·14
95 % CI	0·12, 0·13	0·13, 0·14
Serum lutein/zeaxanthin (μmol/l)		
Geometric mean	0·34	0·34
95 % CI	0·32, 0·35	0·33, 0·35
Serum cholesterol (mmol/l)		
Geometric mean	5·12	5·16
95 % CI	5·07, 5·17	5·11, 5·21
IGF-1 (ng/ml)		
Geometric mean	265	236
95 % CI	260, 271	229, 243
IGFBP-3 (μg/g)		
Geometric mean	4250	4431
95 % CI	4179, 4322	4365, 4497

IGF, insulin-like growth factor; IGFBP, insulin-like growth factor binding protein.

In both sexes, serum IGF-1 and IGFBP-3 concentrations increased with increasing serum concentrations of all carotenoids (Table 2). Statistically significantly positive associations were observed for serum concentrations of lycopene, β-carotene, α-carotene, β-cryptoxanthin and lutein/zeaxanthin with IGF-1 in both men and women (all *P* trend <0·05). Overall, the increase in IGF-1 across quintiles of β-carotene, α-carotene and lutein/zeaxanthin was more pronounced among women than among men, whereas the increase in IGF-1 across quintiles of lycopene and β-cryptoxanthin was more pronounced among men.

**Table 2.** Comparison of serum insulin-like growth factor-1 (IGF-1; ng/ml), serum insulin-like growth factor binding protein-3 (IGFBP-3; μg/g) concentrations and their molar ratio according to categories of serum concentrations of carotenoids and total fruit and vegetable intake among men and women (Geometric means and 95 % confidence intervals)

	IGF-1 (ng/ml)				IGFBP-3 (μg/g)				IGF-1:IGFBP-3 ratio			
	Men		Women		Men		Women		Men		Women	
	Geometric mean*	95 % CI	Geometric mean	95 % CI	Geometric mean	95 % CI	Geometric mean	95 % CI	Geometric mean	95 % CI	Geometric mean	95 % CI
Carotenoids												
Lycopene†												
Q1	254	239, 270	230	216, 245	4113	3951, 4283	4385	4262, 4511	0·22	0·21, 0·23	0·19	0·18, 0·20
Q2	262	251, 273	218	198, 241	4329	4197, 4466	4315	4185, 4449	0·22	0·21, 0·23	0·18	0·17, 0·20
Q3	268	256, 280	234	225, 243	4342	4238, 4448	4430	4325, 4538	0·22	0·21, 0·23	0·19	0·18, 0·20
Q4	262	253, 272	238	227, 249	4250	4127, 4376	4497	4402, 4594	0·22	0·22, 0·23	0·19	0·18, 0·20
Q5	282	273, 292	245	237, 253	4295	4180, 4412	4449	4342, 4560	0·24	0·23, 0·25	0·20	0·19, 0·21
*P* trend	0·01		0·02		0·35		0·07		0·01		0·06	
β-Carotene†												
Q1	252	241, 263	204	180, 232	4289	4150, 4431	4326	4171, 4487	0·21	0·20, 0·22	0·17	0·15, 0·19
Q2	263	254, 272	230	219, 241	4264	4166, 4365	4404	4296, 4515	0·22	0·22, 0·23	0·19	0·18, 0·20
Q3	265	256, 275	234	223, 245	4216	4100, 4335	4401	4285, 4520	0·23	0·22, 0·24	0·19	0·18, 0·20
Q4	274	263, 286	239	227, 252	4288	4171, 4409	4466	4379, 4556	0·23	0·22, 0·24	0·19	0·19, 0·20
Q5	284	273, 295	258	249, 268	4316	4202, 4434	4494	4383, 4608	0·24	0·23, 0·25	0·21	0·20, 0·22
*P* trend	<0·01		<0·01		0·70		0·10		<0·01		0·01	
α-Carotene†												
Q1	256	248, 265	209	186, 235	4209	4108, 4311	4252	4150, 4357	0·22	0·21, 0·23	0·18	0·16, 0·20
Q2	267	256, 279	236	225, 246	4251	4093, 4416	4394	4303, 4487	0·23	0·22, 0·24	0·19	0·19, 0·20
Q3	264	251, 278	226	217, 235	4356	4183, 4535	4334	4201, 4471	0·22	0·21, 0·23	0·19	0·18, 0·20
Q4	267	255, 279	244	232, 256	4216	4116, 4319	4516	4413, 4620	0·23	0·22, 0·24	0·20	0·19, 0·20
Q5	284	271, 298	247	234, 261	4363	4212, 4520	4538	4414, 4666	0·24	0·23, 0·25	0·20	0·19, 0·21
*P* trend	0·01		0·04		0·20		<0·01		0·01		0·19	
β-Cryptoxanthin†												
Q1	257	248, 267	218	201, 237	4154	4055, 4256	4331	4226, 4439	0·22	0·22, 0·23	0·18	0·17, 0·20
Q2	267	258, 276	237	228, 246	4308	4208, 4411	4451	4365, 4538	0·22	0·22, 0·23	0·19	0·18, 0·20
Q3	267	258, 277	243	234, 253	4255	4125, 4389	4452	4347, 4558	0·23	0·22, 0·23	0·20	0·19, 0·21
Q4	262	250, 274	235	223, 248	4271	4161, 4385	4451	4302, 4606	0·22	0·21, 0·23	0·19	0·18, 0·20
Q5	292	281, 304	247	234, 262	4400	4256, 4549	4477	4329, 4629	0·24	0·23, 0·25	0·20	0·19, 0·21
*P* trend	<0·01		0·04		0·06		0·14		0·05		0·07	
Lutein/zeaxanthin†												
Q1	257	245, 269	214	196, 233	4166	4055, 4281	4234	4126, 4345	0·22	0·21, 0·23	0·18	0·17, 0·20
Q2	265	255, 275	233	222, 244	4307	4180, 4437	4402	4290, 4518	0·22	0·21, 0·23	0·19	0·18, 0·20
Q3	273	263, 285	245	235, 256	4274	4180, 4370	4521	4412, 4633	0·23	0·22, 0·24	0·20	0·19, 0·20
Q4	265	252, 279	236	223, 250	4268	4122, 4418	4428	4303, 4557	0·22	0·22, 0·23	0·19	0·18, 0·20
Q5	281	269, 295	244	234, 255	4347	4232, 4465	4540	4419, 4665	0·23	0·23, 0·24	0·19	0·19, 0·20
*P* trend	0·01		0·02		0·10		<0·01		0·07		0·22	
Vegetables†												
Q1	261	251, 271	222	204, 242	4186	4052, 4323	4293	4192, 4397	0·22	0·21, 0·23	0·18	0·17, 0·20
Q2	271	262, 280	237	227, 248	4254	4150, 4362	4372	4255, 4492	0·23	0·22, 0·23	0·19	0·19, 0·20
Q3	261	250, 272	242	232, 252	4305	4186, 4428	4552	4461, 4645	0·21	0·21, 0·22	0·19	0·18, 0·19
Q4	273	258, 289	229	218, 240	4310	4171, 4453	4388	4270, 4510	0·22	0·22, 0·23	0·18	0·17, 0·19
Q5	273	261, 285	241	227, 256	4293	4175, 4415	4501	4392, 4612	0·23	0·22, 0·23	0·19	0·18, 0·20
*P* trend	0·12		0·05		0·15		0·80		0·60		0·79	
Fruits†												
Q1	258	245, 272	229	211, 250	4246	4097, 4400	4333	4224, 4443	0·21	0·21, 0·22	0·19	0·17, 0·20
Q2	266	255, 276	232	224, 241	4228	4122, 4337	4436	4347, 4526	0·22	0·21, 0·23	0·18	0·18, 0·19
Q3	274	263, 286	237	225, 249	4268	4137, 4404	4458	4346, 4573	0·23	0·22, 0·24	0·19	0·18, 0·20
Q4	268	258, 280	234	222, 246	4265	4169, 4363	4433	4340, 4528	0·22	0·22, 0·23	0·19	0·18, 0·20
Q5	271	260, 282	236	225, 248	4368	4247, 4493	4418	4289, 4552	0·22	0·21, 0·23	0·19	0·18, 0·20
*P* trend	0·16		0·34		0·23		0·01		0·50		0·94	

Q, quintile.

* Adjusted for age, BMI, race/ethnicity, smoking, alcohol intake and serum total cholesterol concentration.

† Cutpoints of quintiles are as follows: α-carotene 0·02, 0·06, 0·07, 0·11 µmol/l; β-carotene 0·13, 0·20, 0·32, 0·50 µmol/l; β-cryptoxanthin 0·09, 0·13, 0·18, 0·25 µmol/l; lutein/zeaxanthin 0·23, 0·32, 0·40, 0·53 µmol/l; lycopene 0·22, 0·32, 0·43, 0·56 µmol/l; fruit 6, 14, 30, 44 times per month; vegetables 31, 49, 71, 101 times per month.

Statistically significantly positive associations between serum concentrations of α-carotene and lutein/zeaxanthin with IGFBP-3 were observed in women (all *P* trend <0·05), but not in men. IGFBP-3 concentrations in men and women tended to increase with increasing lycopene, β-carotene and β-cryptoxanthin concentrations, but test for trend was not statistically significant (all *P* trend <0·05).

The IGF-1:IGFBP-3 molar ratio showed statistically significant positive associations with serum concentrations of lycopene, β-carotene and α-carotene in men, but no associations were observed for β-cryptoxanthin and lutein/zeaxanthin. In women, we only observed an association of the IGF-1:IGFBP-3 ratio with β-carotene concentration.

Only fruit intake was significantly positively associated with IGFBP-3 concentrations in women, and there was no significant association in men. Results for IGF-1 concentrations or the IGF-1:IGFBP-3 molar ratio were non-significant in both sexes. The analyses of the association of IGF-1, IGFBP-3 or the IGF-1:IGFBP-3 molar ratio with intake of vegetables did not show any statistically significant finding in women or men (all *P* trend >0·05).

## Discussion

Our study was driven by the observation from animal and cell models that carotenoids, in particular lycopene, might beneficially influence components of the IGF system^(^^29^^)^. Prior to our analysis, twenty epidemiological studies had been published since 1999 that examined possible associations of carotenoid intake or circulating carotenoid concentrations, and consumption of fruits and vegetables with circulating concentrations of IGF-1 and IGFBP-3 (Supplementary Table S1). Of the twenty studies, eleven showed a link between one or more carotenoids, vegetables or fruits and the IGF system with either a positive or negative association. However, the results, as discussed below, were not consistent for carotenoid subgroups or nutrients and partly contradictory.

### Associations with insulin-like growth factor-1

In the NHANES III analysis, higher serum concentrations of carotenoids were associated with higher IGF-1. This is in contrast to the theory that high carotenoid concentrations may protect from cancer by decreasing circulating levels of IGF-1. Our positive associations of serum lycopene concentrations with IGF-1 concentrations in men and women contrast with three other studies (case–control or intervention study), which found an inverse relationship between lycopene (supplement use or serum level) and IGF-1 concentrations^(^^42^^–^^44^^)^ and thirteen studies that did not find any association (see Supplementary Table S1). All three studies with inverse associations had a very small number of participants ranging from twenty to 112 individuals and the two intervention studies^(^^43^^,^^44^^)^ had very short treatment duration of 10–26 d. The positive association between serum lycopene concentrations and IGF-1 concentrations in our analysis might be due to differences in adjustment^(^^42^^,^^44^^)^ or the selection of study participants^(^^43^^)^.

The results of our analysis are in part consistent with those of Suzuki *et al*.^(^^6^^)^, who observed significantly higher serum α-carotene, β-carotene and β-cryptoxanthin with increasing serum IGF-1 concentrations among women, but not men of a Japanese observational study. Our findings for associations with IGF-1 concentrations did not differ by sex. In contrast to our result and that of Suzuki *et al*.^(6)^, one epidemiological study showed that serum β-carotene concentrations were inversely related to IGF-1 concentrations^(^^45^^)^. In addition, positive associations with serum IGF-1 concentrations were found for carotene^(^^46^^)^, α-carotene^(^^6^^,^^47^^)^, β-carotene^(^^6^^)^, β-cryptoxanthin^(^^6^^,^^48^^)^ and for fruit intake^(^^46^^)^, but most other studies (*n* 12; Supplementary Table S1) showed no association with any carotenoid, fruit or vegetable intake and IGF-1.

### Associations with insulin-like growth factor binding protein-3

Positive associations with IGFBP-3 concentrations were found for lycopene^(^^48^^,^^49^^)^, vegetables^(^^50^^)^ and fruits^(^^46^^)^, which is in line with the observation that higher IGFBP-3 concentrations were related to decreased risk of several cancers^(^^51^^–^^53^^)^. In contrast, an inverse relationship with serum IGFBP-3 concentrations was reported in two case–control studies for lycopene, lutein/zeaxanthin^(^^6^^)^ and for vegetable intake^(^^46^^)^. The majority of studies (*n* 12), though, showed no association of any carotenoid examined with IGFBP-3 (Supplementary Table S1). The positive relationships of serum α-carotene and lutein/zeaxanthin concentrations with IGFBP-3 concentrations observed in our analysis in woman are new and in contrast to Suzuki *et al*.^(^^6^^)^, who found an inverse relationship of serum lutein/zeaxanthin concentrations with IGFBP-3 in men. It should be kept in mind that α-carotene concentrations in serum are generally very low and rather variable^(^^54^^)^; thus it cannot be excluded that the observed association is due to chance. Our results are in line with nine studies that reported no significant associations between lycopene and IGFBP-3 (Supplementary Table S1), although also inverse^(^^6^^)^ and positive associations^(^^48^^,^^49^^)^ have been reported.

### Associations with the molar ratio (insulin-like growth factor-1:insulin-like growth factor binding protein-3)

In epidemiological studies, the IGF-1:IGFBP-3 molar ratio has been used as an approximate index of ‘free’, bioactive IGF-1, since IGFBP-3 is the main binding protein of IGF-1 in the circulation^(^^40^^,^^51^^–^^53^^)^. However, the biological effects of the different IGFBP on IGF-1 bioactivity are still relatively unknown. Similar to IGFBP-3, IGFBP-1 and -2 may also reduce bioactive IGF-1 by binding to it and making it unavailable for the IGF-1 receptor. On the other hand, IGFBP-1 and -2 allow the transport of IGF-1 out of the bloodstream, which may result in increased IGF-1 concentrations at the tissue level^(^^55^^)^.

In our analysis, higher molar ratios of IGF-1:IGFBP-3 were related to higher serum concentrations of lycopene, β-carotene and α-carotene in men and to higher β-carotene concentration in women. These findings are in contrast to four other studies^(^^42^^–^^44^^,^^56^^)^ that showed an inverse relationship between lycopene intake or serum concentrations to the molar ratio IGF-1:IGFBP-3; three studies showed no association with the molar ratio (Supplementary Table S1). NHANES III is the first study that examined the molar ratio and its associations with serum concentrations of β-carotene and α-carotene.

### Methodological considerations

This summary of study results shows that a diet rich in carotenoids, vegetables and fruits may influence the IGF system and indirectly cancer risk, but the results were diverse. Study design, study population, and use of circulating concentration of carotenoids or dietary intake may influence the results of a study.

Seven of these previous twenty studies were intervention studies; all others were cross-sectional studies. Intervention studies are considered to provide the most reliable evidence in epidemiological research^(^^57^^)^. However, results of the intervention studies did not differ from cross-sectional studies (see Supplementary Table S1).

The majority of studies were conducted in Europe and in the USA; only a few were conducted in Japan and in Israel. In a Japanese study^(^^6^^,^^46^^)^ both IGF-1 and IGFBP-3 concentrations were positively associated with fruit consumption^(^^44^^)^; in a second Japanese study^(^^10^^)^ lutein/zeaxanthin and IGFBP-3 were inversely associated, and there were no significant results in studies from Europe or the USA. For other carotenoid subgroups and vegetables, we found no differences between European, Asian or US studies.

Of the twenty studies reviewed above, most assessed nutrient intake by FFQ, whereas eight studies measured carotenoid concentration in blood samples. Studies that assessed dietary lycopene intake had similarly inconsistent results as studies that examined circulating lycopene (Supplementary Table S1). Although several studies reported no associations of IGF-1 or IGFBP-3 with either dietary intake or circulating carotenoids (Supplementary Table S1), positive associations with IGF-1 were found for intake of carotenoids^(^^46^^)^ and α-carotene^(^^47^^)^ as well as α- and β-carotene concentration^(^^6^^)^. Negative associations with IGF-1 were found for intake of β-carotene^(^^45^^)^. One study reported an inverse association with IGFBP-3 for lutein/zeaxanthin concentration^(^^6^^)^. In conclusion, it appears that the assessment methodology, i.e., measurement of circulating concentrations or dietary intake assessment, has an impact on the results of a study. To further investigate this potential difference, future studies with both measurement methodologies and comparison of the results will help to elucidate these associations.

Individuals who try to eat a healthy diet, such as high in fruits, vegetables or fish, but low in red meat, sugar or salt^(^^58^^)^, are likely to lead a healthy lifestyle in general. The inability to distinguish the effect of diet from that of other lifestyle factors may pose a threat to the validity of diet–disease associations observed in epidemiological studies^(^^59^^)^. It has been suggested that carotenoids could act as surrogate markers of a diet high in fruits and vegetables^(^^60^^)^, given that high circulating levels are indeed due to high fruit and vegetable consumption rather than supplement intake. Therefore, we investigated the impact of intake of vegetables and fruits on the IGF system in this study as well. Previously, three studies^(^^45^^,^^46^^,^^56^^)^ observed an inverse relationship between vegetable intake and IGF-1, IGFBP-3 and their molar ratio, whereas a fourth^(^^50^^)^ showed positive associations for vegetable intake and IGFBP-3 in African-Americans. The results of NHANES III do not support any of these findings. None of our results for vegetable intake was statistically significant, which is consistent with several other studies (Supplementary Table S1). In contrast to vegetables, fruit intake had a positive association with serum concentrations of IGFBP-3 in women in our analysis. Our findings for the intake of fruits are consistent with those of Maruyama *et al*.^(^^46^^)^, but in contrast to us, they additionally showed positive associations with serum concentrations of IGF-1. Based on the observation that only few associations with the intake of vegetables and fruits but more with the intake of carotenoids were observed, one might conclude that carotenoids do not merely act as surrogate markers of a generally healthy lifestyle. To eliminate confounding, we adjusted for age, race/ethnicity, BMI, cigarette smoking, alcohol consumption and serum total cholesterol concentration.

### Strengths and limitations

The study population is a strength of this study as it is based on a large, nationally representative sample of the US population, and therefore the results have greater external validity than studies of more selected populations^(^^61^^)^. The composition and size of the study population provided us with the opportunity to stratify our analyses by sex. We were able to confirm prior observations regarding the relationship between some carotenoids and the IGF system and showed some new associations especially for lycopene. Diet had been assessed for a 4-week period prior to the interview. Differences in fruit and vegetable availability changes due to seasonality might have affected circulating carotenoids. Degradation of stored samples can be problematic in studies, in which these samples have long, complex storage histories. IGF-1 and IGFBP-3 were measured 10–16 years after the blood samples were obtained^(^^40^^)^. A study by Yu *et al*.^(^^62^^)^ has, however, shown that IGF-1 and IGFBP-3 appear to be stable in response to multiple freeze–thaw cycles. A further limitation of this study is that it is cross-sectional and that our results are based on total circulating IGF-1 levels instead of free IGF-1. Finally, we performed a large number of statistical tests and cannot exclude that some of our finding are simply due to chance.

In conclusion, this analysis further supports that the IGF system, with potential influence on several cancers, may be modified through nutrition, especially carotenoids. In NHANES III, positive relationships between serum concentrations of lycopene, β-carotene, α-carotene, β-cryptoxanthin and lutein/zeaxanthin to IGF-1 concentrations were observed, which is, however, in contrast to the expected inverse associations. The positive associations observed for serum α-carotene and lutein/zeaxanthin concentrations and intake of fruits in relation to IGFBP-3 concentrations are more in line with current thinking of the biological mechanism. Based on ours and the observations of other studies, one might conclude that carotenoids may contribute to IGF-1 and IGFBP-3 modulation, but this very likely depends on the presence of other factors, many of which are still unrevealed or unknown. Clearly, the results deserve confirmation by further larger studies, for example by intervention study to evaluate whether increased consumption of carotenoid-rich fruits and vegetables is able to modulate circulating levels of components of the IGF system.

## Supplementary material

For supplementary material accompanying this paper visit http://dx.doi.org/10.1017/jns.2016.1. click here to view supplementary material
