# Supplementary material for: Associations of serum carotenoid concentrations and fruit or vegetable consumption with serum insulin-like growth factor (IGF)-1 and IGF binding protein-3 concentrations in the Third National Health and Nutrition Examination Survey (NHANES III)
Source: J Nutr Sci. 2016 Mar 8;5:e13. doi: 10.1017/jns.2016.1 (PMC4791518; doi:10.1017/jns.2016.1)
Supplement: Supplementary file 1 [file S204867901600001Xsup001.doc]

Supplementary Table S1. Epidemiological studies of carotenoid, vegetables or fruits intake and association to IGF-1 or IGFBP-3 (1999–2009)

|  | **Author**  **Year**  **Population** | **Study design**  **No. of cases/controls**  **Cancer site** | **Nutrient** | **Major findings: p for trend** | | | | | | | **Comments for results** |
| --- | --- | --- | --- | --- | --- | --- | --- | --- | --- | --- | --- |
| **Observational studies** | | | | | | | | | | | |
| **From blood** | Suzuki K. et al.  2009 (16)  Japan | Case-control within the JACC Study (cohort)  924 controls (578 men, 346 women)  lung and colorectal cancer |  | IGF-1 men | IGFBP-3 men | IGF-1/ IGFBP-3 | IGF-1 women | IGFBP-3 women | IGF-1/ IGFBP-3 | Adjusted for area, age, BMI, serum total cholesterol concentrations, smoking habits, drinking habits and intake amount of energy and protein.  Serum IGF-1 concentrations were higher in the highest quartiles of serum provitamin A such as alpha-carotene, beta-carotene and beta-cryptoxanthin in women.  Serum IGFBP-3 concentrations were negatively associated with serum zeaxanthin/luthein concentrations in men and with serum lycopene concentrations in women. | |
|  |  |  | Beta-carotene | 0.126 | 0.373 |  | **0.018** | 0.425 |  |  |  |
|  |  |  | Alpha-carotene | 0.149 | 0.395 |  | **0.015** | 0.783 |  |  |  |
|  |  |  | Beta-cryptoxanthin | 0.207 | 0.994 |  | **0.018** | 0.669 |  |  |  |
|  |  |  | Lycopene | 0.218 | 0.56 |  | 0.807 | **0.021** |  |  |  |
|  |  |  | Lutein/zeaxanthin | 0.482 | **0.036** |  | 0.317 | 0.278 |  |  |  |
|  | Kim Y.L. et al.  2007 (84)  USA | within CARDIA  470 Black men, 656 White men |  | IGF-1 wh men | IGFBP-3 wh men |  | IGF-1 b men | IGFBP-3 b men |  | Adjusted for age, BMI, alcohol intake, smoking, serum total cholesterol.  Lycopene, sum of four or five carotenoids level were not associated with IGF-1 and IGFBP-3 or their molar ratio in either Black or White men | |
|  |  |  | Lycopene | 0.66 | 0.55 | 0.52 | 0.63 | 0.16 | 0.97 |  |  |
|  |  |  | Sum of four other carotenoids | 0.47 | 0.65 | 0.41 | 0.08 | 0.24 | 0.06 |  |  |
|  |  |  | Sum of all five carotenoids | 0.42 | 0.52 | 0.33 | 0.09 | 0.09 | 0.13 |  |  |
| **From food** | McGreevy K. et al.  2007 (85)  USA | Cross-sectional  95 African American men, 138 white men  prostate cancer |  | IGF-1 wh men | IGFBP-3 wh men |  | IGF-1 AA men | IGFBP-3 AA men |  | Adjusted for age and height.  Increased vegetables intake was positively associated with IGFBP-3 in African Americans while there was no effect in whites. | |
|  |  |  | Fruits, alpha-, beta-carotene, beta-cryptoxanthin | non-significant | non-significant |  | non-significant | non-significant |  |  |  |
|  |  |  | Vegetables | non-significant | 0.87 |  | non-significant | **0.03** |  |  |  |
|  | Norat T. et al.  2007 (86)  Europe | Case-control within EPIC  2109 women  breast cancer |  |  |  |  | IGF-1 women | IGFBP-3 women |  | Adjustment for total energy intake, age, BMI, physical activity, smoking, centre and laboratory batch.  Vegetables and beta-carotene was negatively associated with IGF-1. | |
|  |  |  | Fruits |  |  |  | 0.06 | 0.24 |  |  |  |
|  |  |  | Vegetables |  |  |  | **0.02** | 0.74 |  |  |  |
|  |  |  | Beta-carotene |  |  |  | **0.02** | 0.55 |  |  |  |
| **From food** | Tran C.D. et al.  2006 (87)  USA | 1542 healthy women |  |  |  |  | IGF-1 women | IGFBP-3 women |  | Adjusted for menopausal status, age, BMI, physical activity, energy intake, hormone replacement therapy, oral contraceptives, duration of lactation, no. of pregnancies, age at menarche, alcohol intake, smoking.  Concentrations of growth factors were not associated with total fruits or total vegetables.  No associations between intakes of b-carotene or lycopene from food, supplements, or both. | |
|  |  |  | Vegetables |  |  |  | 0.62 | 0.77 |  |  |  |
|  |  |  | Fruits |  |  |  | 0.15 | 0.18 |  |  |  |
|  |  |  | Fruits and vegetables |  |  |  | 0.67 | 0.74 |  |  |  |
|  |  |  | Beta-carotene |  |  |  | 0.9 | 0.46 |  |  |  |
|  |  |  | Lycopene |  |  |  | 0.4 | 0.81 |  |  |  |
|  | Holmes M.D. et al.  2002 (88)  USA | Case-control within the NIIS  1037 healthy women |  |  |  |  | IGF-1 women | IGFBP-3 women |  | Adjusted for energy intake, age, laboratory batch, menopausal status, duration of hormone replacement, smoking, physical activity, parity, lactation, BMI.  Beta-cryptoxanthin was positively associated with IGF-1. Lycopene intake was positively associated with IGFBP-3. | |
|  |  |  | Carotene |  |  |  | 0.08 | 0.27 |  |  |  |
|  |  |  | Alpha-carotene |  |  |  | 0.85 | 0.87 |  |  |  |
|  |  |  | Beta-carotene |  |  |  | 0.15 | 0.34 |  |  |  |
|  |  |  | Beta-cryptoxanthin |  |  |  | **0.03** | 0.52 |  |  |  |
|  |  |  | Lutein/Zeaxanthin |  |  |  | 0.34 | 0.66 |  |  |  |
|  |  |  | Fruits |  |  |  | 0.58 | 0.49 |  |  |  |
|  |  |  | Vegetables |  |  |  | 0.9 | 0.55 |  |  |  |
|  |  |  | Lycopene |  |  |  | 0.5 | **0.003** |  |  |  |
|  | Vrieling A. et al.  2004 (89)  Netherlands | within the EPIC  386 women |  | IGF-1 pm women | IGFBP-3 pm women |  | IGF-1 women | IGFBP-3 women |  | Adjusted for age, BMI, physical activity score, time since last meal or drink, and total energy intake.  For intake of lycopene no statistically significant associations were found with plasma component of the IGF-system. | |
|  |  |  | Lycopene | 0.89 | 0.77 |  | 0.9 | 0.47 |  |  |  |
|  | Maruyama K. et al.  2009 (90)  Japan | within JACC  10350 controls |  | IGF-1 men | IGFBP-3 men |  | IGF-1 women | IGFBP-3 women |  | Adjustment for age.  IGF-1 and IGFBP-3 were positive associated with higher intakes of fruits. IGFBP-3 were negative associated with higher intakes of vegetables. IGF-1 were positive associated with higher intakes of carotenoids in women. | |
|  |  |  | Carotene | 0.78 | 0.29 |  | **0.02** | 0.61 |  |  |  |
|  |  |  | Tomatoes | 0.73 | 0.49 |  | 0.16 | 0.24 |  |  |  |
|  |  |  | Vegetables | 0.83 | **0.001** |  | 0.46 | **0.04** |  |  |  |
|  |  |  | Fruits | **<0.001** | **<0.001** |  | **<0.001** | **<0.001** |  |  |  |

| **From food** | Maskarinec G. et al.  2005 (91)  USA (Hawaii) | | 258 women |  |  |  |  | IGF-1 women | | IGFBP-3 women | IGF-1/ IGFBP-3 women | | Adjusted for age, ethnicity, BMI, laboratory batch.  Weak trends of higher IGF-1 with higher intake of fruits, not statistically significant. | |
| --- | --- | --- | --- | --- | --- | --- | --- | --- | --- | --- | --- | --- | --- | --- |
|  |  |  |  | Vegetables |  |  |  | 0.32 | | 0.83 | 0.50 | |  |  |
|  |  |  |  | Fruits |  |  |  | 0.17 | | 0.69 | 0.44 | |  |  |
|  | Signorello L.B. et al.  2000 (92)  Greece | | Case-control  153 men |  | IGF-1 men |  |  |  | |  |  | | Adjusted for age, height, BMI, smoking, alcohol and coffee intake, total energy intake.  No nutritional variable was significantly related to serum IGF-1 concentrations. | |
|  |  |  |  | Tomatoes | non-significant |  |  |  |  |  |  | |  |  |
|  | Kaklamani V.G. et al.  1999 (93)  Greece | | 115 men and women  healthy |  | IGF-1 | IGFBP-3 |  |  | |  |  | | Adjustment for total energy intake, age, sex, height, BMI, smoking, alcohol, coffee.  Serum IGF-1 and IGFBP-3 concentrations are not significantly associated with vegetables consumption. | |
|  |  |  |  | Vegetables | non-significant | non-significant |  |  |  |  |  | |  |  |
|  | Gunnell D. et al.  2003 (94)  USA | | Case-control  368 men  healthy |  | IGF-1 men | IGFBP-3 men | IGF-1/ IGFBP-3 men |  | |  |  | | Age-, centre- and energy-adjusted.  IGF-1 tended to be lower and IGFBP-3 higher in those who ate tomatoes or toamto-containing products more frequently, although evidence for a trend was only clear for IGF-1/IGFBP-3 molar ratio.  The molar ratio was inversely related to vegetable intake. | |
|  |  |  |  | Vegetables | 0.5 | 0.22 | **0.045** |  |  |  |  | |  |  |
|  |  |  |  | Tomatoes | 0.19 | 0.7 | 0.28 |  |  |  |  | |  |  |
|  |  |  |  | Baked beans | 0.55 | 0.63 | 0.64 |  |  |  |  | |  |  |
|  |  |  |  | Tomato ketchup | 0.1 | 0.09 | **0.005** |  |  |  |  | |  |  |
|  |  |  |  | Tomato juice | 0.14 | 0.47 | **0.004** |  |  |  |  | |  |  |
|  | Mucci L.A. et al.  2001 (95)  Greece | | Case-control  112 men |  | IGF-1 men |  | IGF-1/ IGFBP-3 men |  | |  |  | | Adjustment for age, alcohol, total energy.  Significantly inversely associated with IGF-1 concentrations and IGF-1/IGFBP-3 molar ratio. | |
|  |  |  |  | Cooked tomatoes | **0.014** |  | **0.047** |  |  |  |  | |  |  |
| **Intervention studies** | | | | | | | | | | | | | | |
| **From blood** | | Riso P. et al.  2006 (96)  Italy | Placebo controlled crossover study over 26 days  20 young persons  healthy |  | IGF-1 | IGFBP-3 | IGF-1/ IGFBP-3 | |  |  | |  | | Increases in lycopene concentrations were inversely correlated with those in serum IGF-1; IGFBP-3 concentrations were not affected by lycopene supplementation  The changes in lycopene concentration were inversely and significantly correlated with those of IGF-1 (p=0.03) and IGF-1/IGFBP-3 ratio (p<0.05). No relationship was apparent between plasma carotenoid concentrations and IGFBP-3 changes. |
|  |  |  |  | Lycopene | **0.03** |  | **<0.05** | |  |  |  |  | |  |

| **From blood** | Graydon R. et al.  2007 (97)  Northern Ireland | Double-blind placebo-controlled  20 men  healthy |  | IGF-1 men | IGFBP-3 men | IGF-1/ IGFBP-3 men |  |  |  | No significant effect of lycopene supplementation on IGF-1 or IGFBP-3 in healthy male. |
| --- | --- | --- | --- | --- | --- | --- | --- | --- | --- | --- |
|  |  |  | Lycopene | 0.52 | 0.55 | 0.60 |  |  |  |  |
|  | Graydon R. et al.  2007 (98)  Northern Ireland | 54 men  healthy |  | IGF-1 men |  |  |  |  |  | IGF-1 was significantly positively associated with serum concentrations of alpha-carotene. No association between lycopene concentration and IGF-1 concentration. |
|  |  |  | Lycopene | 0.1 |  |  |  |  |  |  |
|  |  |  | Alpha-carotene | **0.01** |  |  |  |  |  |  |
|  |  |  | Beta-carotene | 0.87 |  |  |  |  |  |  |
|  | Walfisch S. et al.  2007 (99)  Israel | Double-blind randomized trial, Tomato-oleoresin treatment  78  colorectal cancer |  | IGF-1 | IGFBP-3 | IGF-1/ IGFBP-3 |  |  |  | Supplementation with tomato carotenoids caused a significant 25% drop in IGF-1 blood concentration after 10 days. No change in IGFBP-3. The IGF-1/IGFBP-3 molar ratio was significantly decreased. |
|  |  |  | Lycopene | **0.02** | non-significant | **0.03** |  |  |  |  |
|  | Vrieling A. et al.  2007 (100)  Netherlands | Randomized, placebo-controlled, double-blinded crossover study  40 men and 31 postmenopausal women  colorectal cancer |  | IGF-1 men | IGFBP-3 men |  | IGF-1 pm women | IGFBP-3 pm women |  | Lycopene supplementation did not significantly affect serum total IGF-1 concentrations in men or women.  Serum IGFBP-3 concentrations were not significantly affected by lycopene supplementation in men; positive association between relative changes in lycopene concentrations and relative changes in serum IGFBP-3 in women. |
|  |  |  | Lycopene | non-significant | non-significant |  | 0.08 | **<0.01** |  |  |
|  | Kucuk O. et al.  2001 (101)  USA | Randomized lycopene supplementation for 3 w  13 men, 10 controls  prostate cancer |  | IGF-1 men | IGFBP-3 men |  |  |  |  | IGF-1 and IGFBP-3 decreased in both intervention and control group. |
|  |  |  | Lycopene | 0.88 | 0.49 |  |  |  |  |  |
| **From food** | Flood A. et al.  2008 (102)  USA | Randomized trial of a dietary intervention within Polyp Prevention Trial  248 Intervention, 302 controls  colorectal cancer |  | IGF-1 | IGFBP-3 |  |  |  |  | Dietary intervention did not affect the relative rate of decline in IGF-1 or IGFBP-3 after 4 years. |
|  |  |  | High fruits, -vegetables, -fibre, low fat diet | 0.61 | 0.81 |  |  |  |  |  |
